# Supplementary material for: MAP3Kε1/2 Interact with MOB1A/1B and Play Important Roles in Control of Pollen Germination through Crosstalk with JA Signaling in Arabidopsis
Source: Int J Mol Sci. 2022 Feb 28;23(5):2683. doi: 10.3390/ijms23052683 (PMC8910673; doi:10.3390/ijms23052683)
Supplement: Supplementary file 1 [file ijms-23-02683-s001.zip › ijms-1597632-supplementary.pdf]

Figure. S1

A

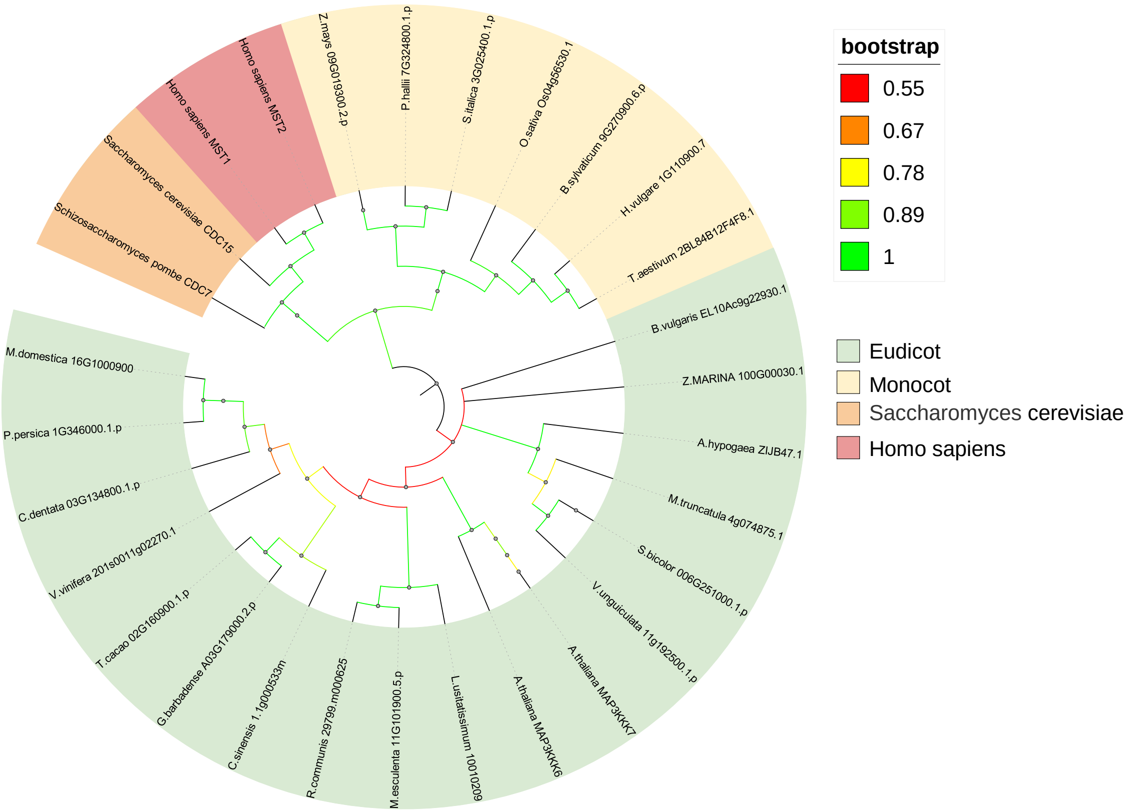

B

STKc_Cdc7_like

```
                                  #####      #    # #      #                  ####     ##     #       # ####   #
MAP3Kε1| Arabidopsis                22 LGDEIGKGAYGRV----AIK---NIVKYLGSS-TKTHLHIILEYVENGSLANI---HRDIKGANIL 151
MAP3Kε2| Arabidopsis                22 LGDEIGKGAYGRV----AIK---NIVKYLGSL-TKTHLHIILEYVENGSLANI---HRDIKGANIL 151
ZmPHJ40.09G019300.2.p| Z.mays PHJ   22 LGDEIGKGAYGRV----AIK---NIVKYLGSL-TKSHLHIILEYVENGSLANI---HRDIKGANIL 151
LOC_Os04g56530.1|O.sativa           22 LGDEIGKGAYGRV----AIK---NIVKYLGSL-TRSHLHIILEYVENGSLANI---HRDIKGANIL 151
 Glyma.11G101700.4.p|G.maxWm        22 LGDEIGKGAYGRV----AIK---NIVKYLGSS-KSHLHIVLEYVENGS LANI---HRDIKGANIL 151
CDC15 |Saccharomyces cerevisiae     27 LKQVIGRGSYGVV----AIK---NIVKYHGFI-KSYELYILLEYCANGSLRRL---HRDIKAANIL 153
CDC7 |Schizosaccharomyces pombe     11 LGDCLGKGAFGAV---AVK---NIVKYRGSY--NDSLCIILEYCENGSLRSI---HRDIKGANIL 138
                                          ##     #              ####   #              #            #
MAP3Kε1| Arabidopsis               152 TTKEGLVKLADFGVATKL---NTHSVVGTPYWMAPE----TVIELLTCVPPYYDLQPMP     226
MAP3Kε2| Arabidopsis               152 TTKEGLVKLADFGVATKL---NTHSVVGTPYWMAPE---- TIIELLTCVPPYYDLQPMP    226
ZmPHJ40.09G019300.2.p| Z.mays PHJ  152 TTKEGLVKLADFGVATKL---NTHSVVGTPYWMAPE----TVIELLTCVPPYYDLQPMP     226
LOC_Os04g56530.1|O.sativa          152 TTKEGLVKLADFGVATKL---NTHSVVGTPYWMAPE----TVIELLTCAPPYYDLQPMP     226
Glyma.11G101700.4.p|G.maxWm        152 TTKEGLVKLADFGVATKL---NTHSVVGTPYWMAPE----TVIELLTCVPPYYDLQPMP     226
CDC15 |Saccharomyces cerevisiae    154 LSADNTVKLADFGVSTIVNSSALTLAGTLNWMAPE---TVVEMLTKNPPYHNLTDAN      225
 CDC7 |Schizosaccharomyces pombe   139  TTKDGTIKLADFGVATKI---EDHSVVGSPYWMAPE----TVIELLDGNPPYYDLDPTS    211
```

Figure S1. A phylogenetic analysis of the MAP3Kε family. **A:** Phylogenetic relationships of members of the MAP3Kε family. Rooted circular phylogram representation of the consensus tree generated by the neighbor-joining method (500 bootstraps) on the conserved regions of proteins from the MAP3Kε family. Triangle represents NJ bootstrap values > 50. numbers after the abbreviated species names are internal. The aqua represents eudicot, the buff represents monocot, salmon pink represents *Saccharomyces cerevisiae*, the rose represents *Homo sapiens.* The bootstrap represents similarity, rendered with a color gradient. **B:** The conserved regions of MAP3Kε from Arabidopsis thaliana and examples from other representative taxa.

Figure. S2

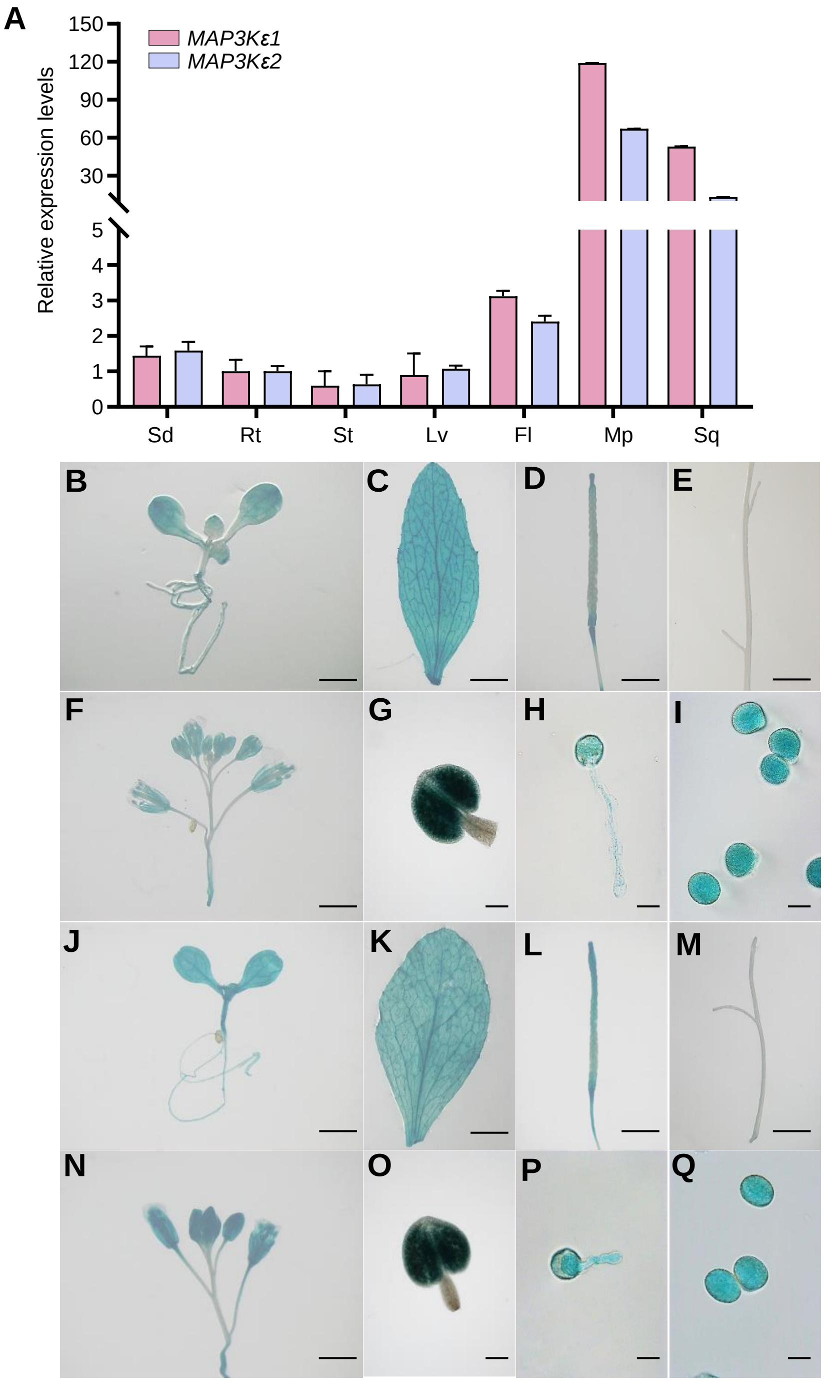

Figure S2. Expression patterns and levels of *MAP3Kε1* and *MAP3Kε2.* **A:** Quantitative real-time PCR showing the expression levels of the *MAP3Kε1* and *MAP3Kε2* in different tissues from Arabidopsis. Sd, seedlings; Rt, root; St, steam; Lv, leaves; Fl, flower; Mp, mature; Sq, silique. Each bar represents the mean ± SEM of three independent experiments. **B-I:** The *GUS* expression patterns of p*MAP3Kε1*::*GUS* transgenic lines. **J-Q:** The *GUS* expression patterns of p*MAP3Kε2*::*GUS* transgenic lines. Seedings (**B** ,**J**); leaves (**C**, **K**); siliques(**D**, **L**); steams (**E**, **M**); inflorescences (**F**, **N**); anthers (**G**, **O**); pollen tubes (**H**, **P**); mature pollens (**I**, **Q**). The scale bars : 2 mm for (**B**-**F**) and (**J**-**N**), 100 μm for (**G**-**I**) and (**P**-**Q**).

Figure. S3

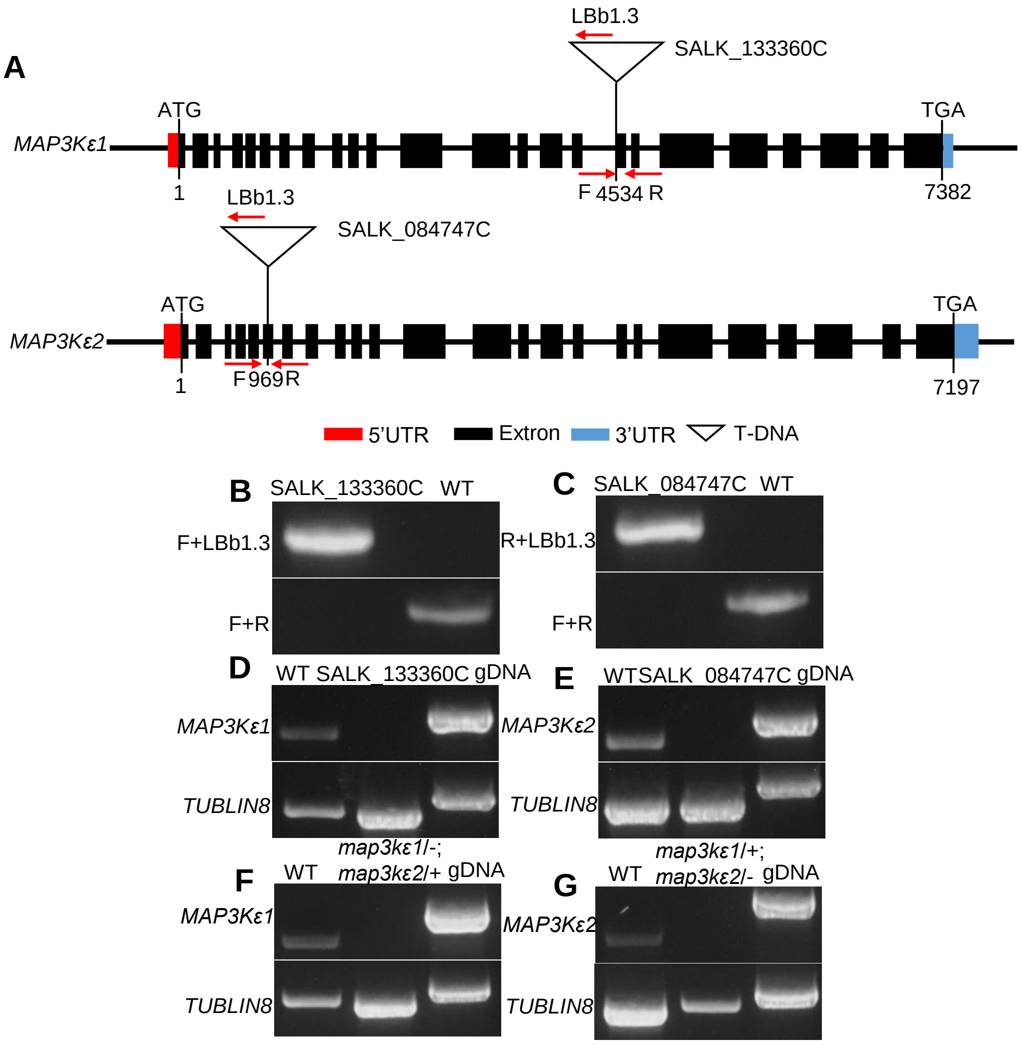

Figure S3. Molecular characterization of *map3kε1*, *map3kε2* mutants. **A:** The schematic diagrams of the *MAP3Kε1*, *MAP3Kε2* structures and T-DNA insertion sites in the mutants. Red arrows represent primers. **B:** Confirmation of the T-DNA insertion in *map3kε1* by PCR. **C:** Confirmation of the T-DNA insertion in *map3kε2* by PCR. **D:** The relative expression levels of *MAP3Kε1* genes in wild-type (WT) and SALK_1333690C mutant by RT-PCR. **E:** The relative expression levels of *MAP3Kε2* genes in wild-type (WT) and SALK_084747C mutant by RT-PCR. **F:** The relative expression levels of *MAP3Kε1* genes in wild-type (WT) and *map3kε1*/-; *map3kε2*/+ mutant by RT-PCR. **G:** The relative expression levels of *MAP3Kε2* genes in wild-type (WT) and *map3kε1*/+; *map3kε2*/- mutant by RT-PCR. *TUBLIN8* used for control.

Figure. S4

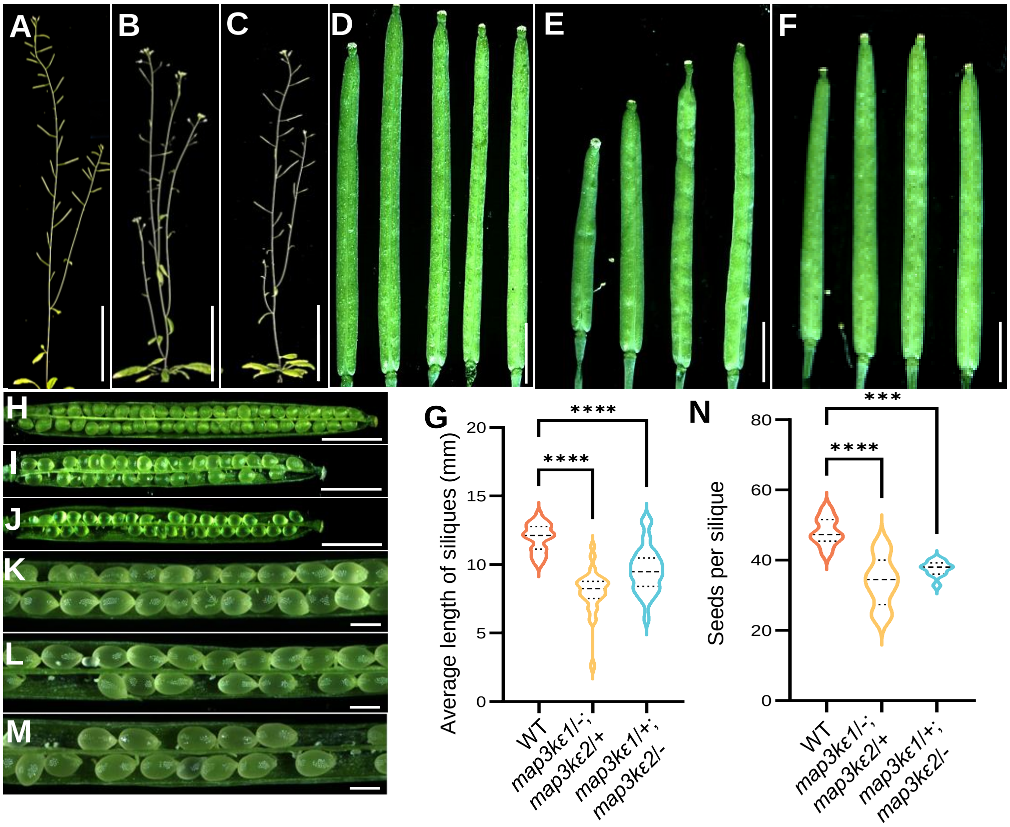

Figure S4. The *map3kε1*/-; *map3kε2*/+ and *map3kε1*/+; *map3kε2*/- mutants reduced fertility. **A-C:** The vegetative and reproductive growth patterns of wild-type (WT) (**A**) and the *map3kε1*/-; *map3kε2*/+ (**B**) and *map3kε1*/+; *map3kε2*/-(**C**) mutant plants. **D-F:** The siliques length from wild-type (WT) plants (**D**), *map3kε1*/-; *map3kε2*/+ (**E**) plants and *map3kε1*/+; *map3kε2*/- (**F**) plants. **G:** Comparison silique length in wild-type (WT) , *map3kε1*/-; *map3kε2*/+ and *map3kε*1/+; *map3kε2*/-. **H-M:** The silique with seed set from wild-type (WT) (**H**, **K**) and the *map3kε1*/-; *map3kε2*/+ (**I**, **L**) and *map3kε1*/+; *map3kε2*/- (**J**, **M**) mutant plants. **N:** Comparison seeds per silique in wild-type (WT) , *map3kε1*/-; *map3kε2*/+ and *map3kε1*/+; *map3kε2*/-, each bar represents the mean ± SD of three independent experiments. Different letters represent significant difference at ***p < 0.001, ****p < 0.0001 (one-way ANOVA, Tukey post-test). Bars = 1mm in (**K**-**M**), Bars = 2 mm in (**D**-**J**), Bar=5 cm in (**A**-**C**).

Figure. S5

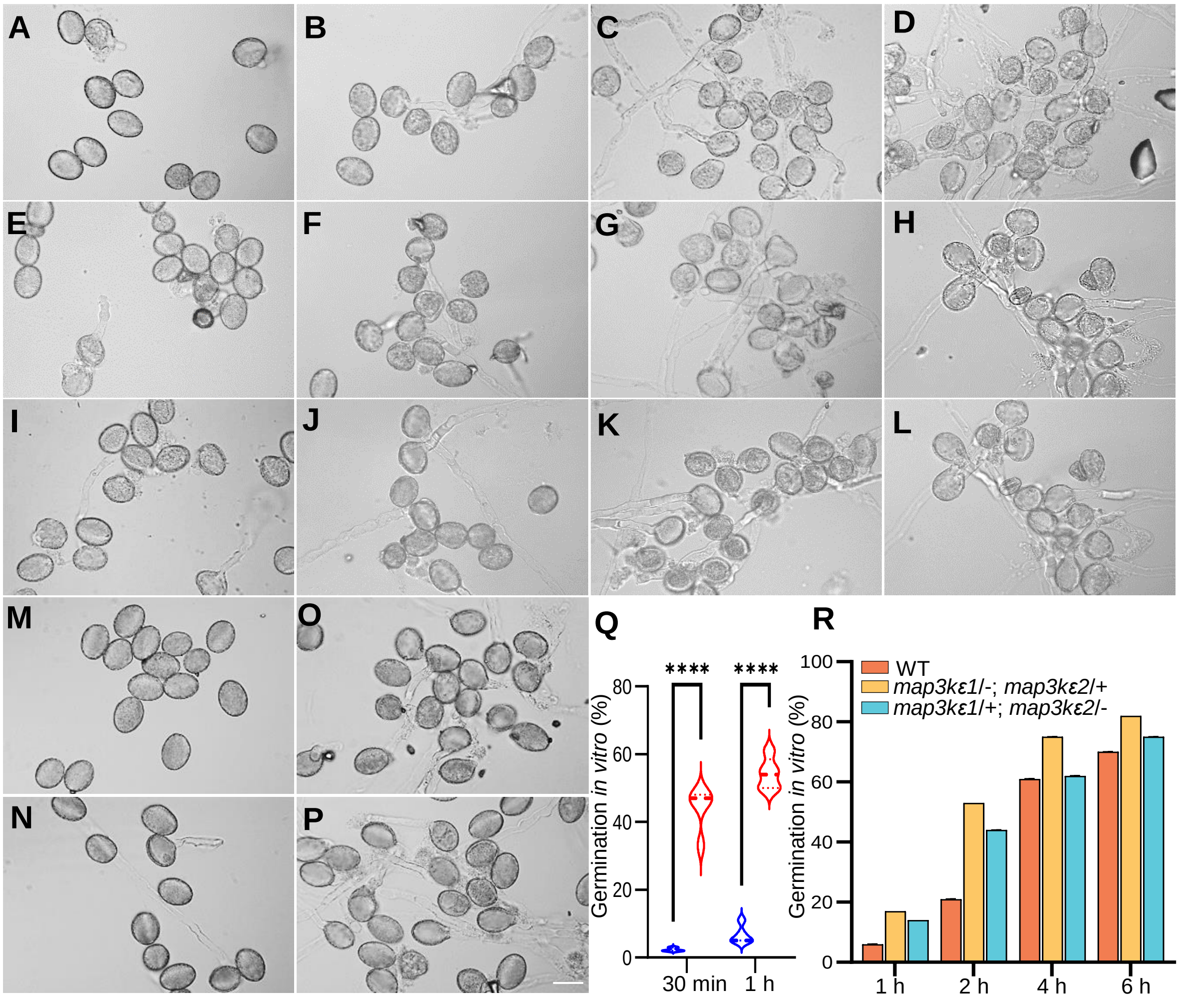

Figure S5. Arabidopsis pollen germinated *in vitro*. **A-D:** The wild-type (WT) pollen grains germinated *in vitro* at 1h, 2h, 4h, 6h respectively. **E-H:** The *map3kε1*/-; *map3kε2*/+ pollen grains germinated *in vitro* at 1h, 2h, 4h, 6h respectively. **I-L:** The *map3kε1*/+; *map3kε2*/- pollen grains germinated *in vitro* at 1h, 2h, 4h, 6h respectively. **M-N:** The wild-type (WT) pollen grains germinated *in vitro* with the most germination on the medium supplemented with 0 μM MeJA at 30 min and 1h, respectively. **O-P:** The wild-type (WT) pollen grains germinated *in vitro* with the most germination on the medium supplemented with 100 μM MeJA at 30 min and 1h, respectively. **Q:** Germination of wild-type (WT) pollen in standard germination medium supplemented with MeJA. **R:** Comparison pollen germination in wild-type (WT) , *map3kε1*/-; *map3kε2*/+ and *map3kε1*/+; *map3kε2*/-, each bar represents the mean ± SD of three independent experiments. Different letters represent significant difference at ****p < 0.0001 (one-way ANOVA, Tukey post-test). Bars = 20 μm (**A-P**).

Figure. S6

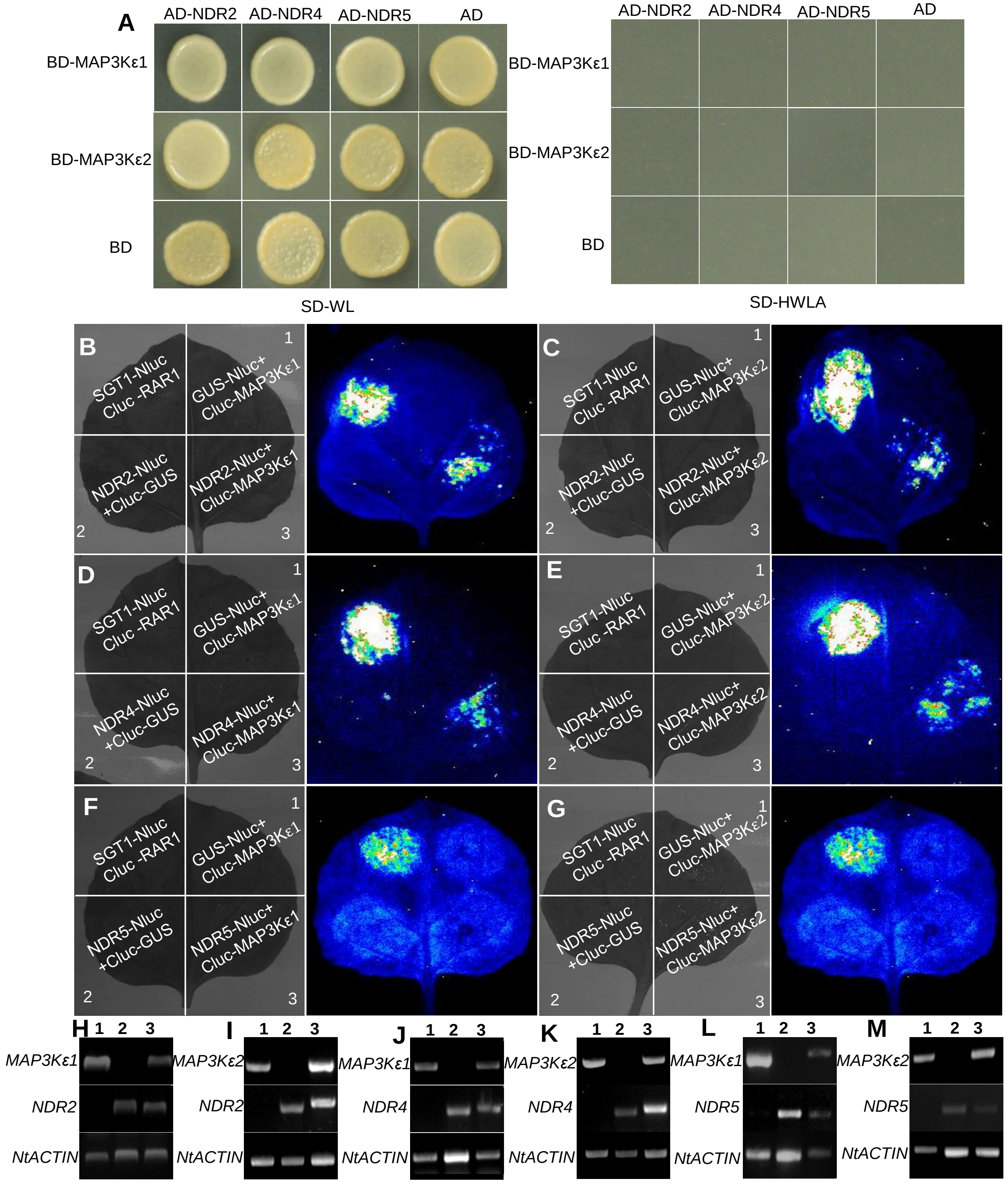

A
AD-NDR2
AD-NDR4
AD-NDR5
AD
BD-MAP3Kε1
BD-MAP3Kε2
BD
SD-WL
SD-HWLA
B
C
D
E
F
G
SGT1-Nluc Cluc -RAR1
GUS-Nluc+ Cluc-MAP3Kε1
GUS-Nluc+ Cluc-MAP3Kε2
NDR2-Nluc +Cluc-GUS
NDR2-Nluc+ Cluc-MAP3Kε1
NDR2-Nluc+ Cluc-MAP3Kε2
NDR4-Nluc +Cluc-GUS
NDR4-Nluc+ Cluc-MAP3Kε1
NDR4-Nluc+ Cluc-MAP3Kε2
NDR5-Nluc +Cluc-GUS
NDR5-Nluc+ Cluc-MAP3Kε1
NDR5-Nluc+ Cluc-MAP3Kε2
1
2
3
H
I
J
K
L
M
MAP3Kε1
MAP3Kε2
NDR2
NDR4
NDR5
NtACTIN

Figure S6. Yeast two-hybrid assay and LCI assay for MAP3Kε1/2 protein and NDR2/4/5. **A:** MAP3Kε1/ 2 can't interact with MOB in the yeast two-hybrid assay. Interaction was determined by yeast growth on the medium lacking His (H), Trp (W) , Leu (L) and Ade(A). **B:** MAP3Kε1 interacts with NDR2 in the luciferase complementation image (LCI) assays. **C:** MAP3Kε2 interacts with NDR2 in the LCI assay. **D:** MAP3Kε1 interacts with NDR4 in the LCI assay. **E:** MAP3Kε2 interacts with NDR4 in the LCI assay. **F:** MAP3Kε1 can't interact with NDR5 in the LCI assay. **G:** MAP3Kε2 can't interact with NDR5 in the LCI assay. **H:** RT-PCR analysis for gene expression in the LCI assay of (**B**). The numbers represent different experimental combinations marked in (**B**). **I:** RT-PCR analysis for gene expression in the LCI assay of (**C**). The numbers represent different experimental combinations marked in (**C**). **J:** RT-PCR analysis for gene expression in the LCI assay of (**D**). The numbers represent different experimental combinations marked in (**D**). **K:** RT-PCR analysis for gene expression in the LCI assay of (**E**). The numbers represent different experimental combinations marked in (**E**). **L:** RT-PCR analysis for gene expression in the LCI assay of (**F**). The numbers represent different experimental combinations marked in (**F**). **M:** RT-PCR analysis for gene expression in the LCI assay of (**G**). The numbers represent different experimental combinations marked in (**G**). The expression of *Nicotiana benthamiana* gene *ACTIN* as a control for the internal standard.

Figure. S7

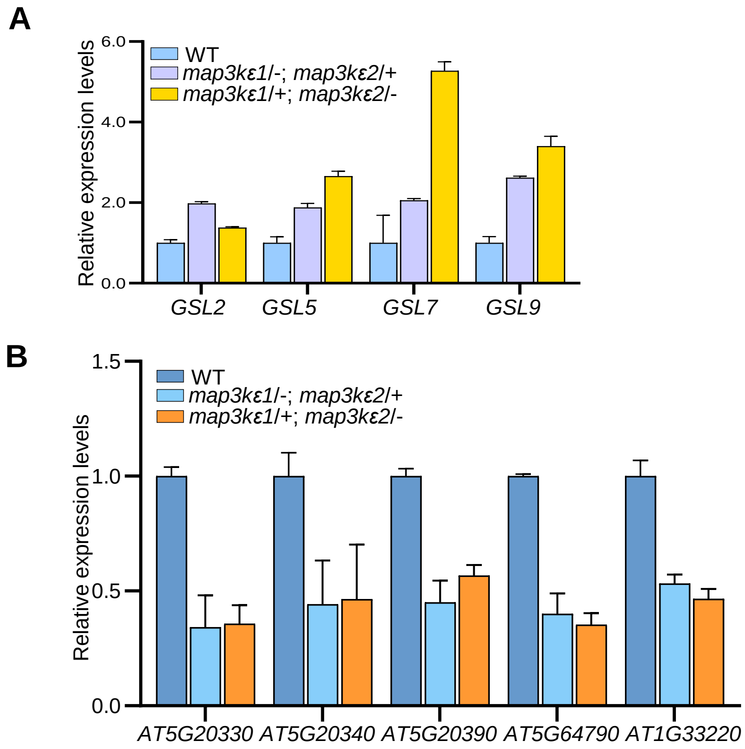

Figure S8. Expression of callose synthase and 1,3-glucanase genes were altered in the *map3kε1*/-; *map3kε2*/+ and *map3kε1*/+; *map3kε2*/- mutant. **A:** Relative expression of *GSL2*, *GSL5*, *GSL7*, *GSL9* in mature pollen of wild-type (WT) and the *map3kε1*/-; *map3kε2*/+ and *map3kε1*/+; *map3kε2*/- mutant plants. **B:** Relative expression of *AT5G20330* (β-1,3-glucanase), *AT5G20340* (β-1,3-glucanase), *AT5G20390* (glycosyl hydrolase), *AT5G64790* (o-glycosyl hydrolases), *AT1G33220* (glycosyl hydrolase) in mature pollen of wild type (WT) and the *map3kε1*/-; *map3kε2*/+ and *map3kε1*/+; *map3kε2*/- mutant plants. *ACTIN2* was used as an internal control.

Table S1. The sequences of the primers used in this study

Uses of the primers	Names of the primers	Sequences of the primers (5'-3')
Mutant identification	LBb1.3	ATTTTGCCGATTTCGGAAC
	GK-LB	ATATTGACCATCATACTCATTGC
	GK-719G04-LP	TGGATTCAGGTTCATGGTTGA
	GK-719G04-RP	TGCACTCTTCTTAGGTCGGA
	SALK_062070-LP-2	TTTGGAGGAAAAGCTGCACC
	SALK_062070-RP-2	ACTGCTGGTCCCAAGTACAG
	SALK_133360-F	GCAGGATTTTTGTTGTTGTCC
	SALK_133360-R	AATCATTTCTTGGGGTGGATC
	SALK_084747-F	TGGAAAACATTGGTCAAGAGG
	SALK_084747-R	AGGAATAGGAGGGGTATCATC
Complementation assays	MAP3Kε1-gus-cz-F((PstI)	AAGCTTGCATGCCTGCAGCCGTGTTCACCACCAAAG
	MAP3Kε1-gus-cz-R((BamHI)	CTGACCACCCGGGGATCCctcttctcgtttcttctt
	MAP3Kε2-GUS-F(PstI):	GCTGCAGCTGTTCTCTGATCACCGGTTG
	MAP3Kε2-GUS-R(BamHI)	CGGATCCctctttctcgtcagcaccaaac
	pMAP3Kε1-1300-F (PstI)	agtgccaagcttCTGCAGCCGTGTTCACCACCAAAG
	pMAP3Kε1-1300-R (BamHI)	CACCATGGTACCGGATCCctcttctcgtttcttctt

	1300-MAP3Kε1-F(Sac I)	GAGCTGTACAAGGAGCTCATGGCGCGGCAAATGACG
	1300-MAP3Kε1-R(Sac I)	GAGCTGTACAAGGAGCTCATGGCGCGGCAAATGACG
	pMAP3Kε2-1300-F (PstI)	agtgccaagcttCTGCAG CTGTTCTCTGATCACCGG
	pMAP3Kε2-1300-R (BamHI)	CACCATGGTACCGGATCCctctttctcgtcagcacc
	1300-MAP3Kε2-F(Sac I)	GAGCTGTACAAGGAGCTCATGGCGCGACAGATGACG
	1300-MAP3Kε2-R(Sac I)	TCGGGGAAATTCGAGCTCTCACAAGATGGTGTTGAT
Y2H assays	MAP3Kε1-AD-F(NdeI)	CCAGATTACGCTCATATGATGGCGCGGCAAATGACG
	MAP3Kε1-AD-R(EcoRI)	CCCACCCGGGTGGAATTCTCACAATATTGTGTTGAT
	MAP3Kε2-AD-F(NdeI)	CCAGATTACGCTCATATGATGGCGCGACAGATGACG
	MAP3Kε2-AD-R(EcoRI)	CCCACCCGGGTGGAATTCTCACAAGATGGTGTTGAT
	MOB1A-CDS-BD-F(EcoRI_	CCGGAATTCATGAGTCTCTTTGGGTTAGG
	MOB1A-CDS-BD-R(BamHI)	CGCGGATCCTCAATAAGGTGAAATGATAG
	MOB1B-CDS-BD-F(EcoRI_	CCGGAATTCATGAGTCTATTCGGGCTTGG
	MOB1B-CDS-BD-R(BamHI)	CGCGGATCCTTAGTAAGGTGCAATGATAG
CoIP assays	1390-MAP3Kε1-gfp-F ((BamHI)	GGGATCCATGGCGCGGCAAATGACGTC

	1390-MAP3Kε1-gfp-R（KpnI）	GGGTACCCAATATTGTGTTGATGTGAAG
	1390-MAP3Kε2-gfp-F（SalI）	GGTCGACATGGCGCGACAGATGACGTC
	1390-MAP3Kε2-gfp-R（KpnI）	GGGTACCCAAGATGGTGTTGATGTGAAG
LCI assays	MAP3Kε1-cluc-F（KpnI）	GGGTACCATGGCGCGGCAAATGACGTC
	MAP3Kε1-cluc-R（BamHI）	GGGATCCTCACAATATTGTGTTGATGTG
	MAP3Kε2-cluc-F(KpnI）	GGGTACCATGGCGCGACAGATGACGTC
	MAP3Kε2-cluc-R（SalI）	GGTCGACTCACAAGATGGTGTTGATGT
	MOB1A-nluc-F(KpnI)	GGGTACCATGAGTCTCTTTGGGTTAGG
	MOB1A-nluc-R(SalI）	GGTCGACATAAGGTGAAATGATAGATTCT
	MOB1B-nluc-F（KpnI）	GGGTACCATGAGTCTATTCGGGCTTGG
	MOB1B-nluc-R（SalI）	GGTCGACGTAAGGTGCAATGATAGATTC
RT-PCR	NtACTIN-F	CCACACAGGTGTGATGGTTG
	NtACTIN-R	GTGGCTAACACCATCACCAG
	MAP3Kε1-RT-F	CTGAGAGGGATCGTTCTCGC
	MAP4Kε1-RT-R	GGGTGCAAAGCATCTGGTTG
	MAP3Kε2-RT-F	TGGAAAACATTGGTCAAGAGG
	MAP3Kε2-RT-R	AGGAATAGGAGGGGTATCATC
	MOB1A-RT-F	AGAGTGCACCTTCTGGAAGC
	MOB1A-RT-R	ATGAGCTCTTGTAGAGGCGC
	MOB1B-RT-F	TTCCGTCCTAAGAAGAGCGC
	MOB1B-RT-R	AGGAGCGAGTTCTTTCTTGTCA

qRT-PCR	ACT2-qPCR-FP	GATGCCCAGAAGTCTTGTTCC
	ACT2-qPCR-RP	TGCTCATACGGTCAGCGATAC
	LOX3-qPCR-FP	GAAATCAATGCTTTGGCTAG
	LOX3-qPCR-RP1	AAGACCGTCGTTGGCGTA
	LOX4-qPCR-FP	TGCTTCACTGCTGGTCAATA
	LOX4-qPCR-RP1	ACCGTCGTTGGCGTATGG
	LOX6-qPCR-FP	TCCTGCTGATCTTGTTCG
	LOX6-qPCR-RP1	GCTCAAGGTCAGATGTTATG
	AOS-qPCR-FP	CGGTTACGGCTCAATACG
	AOS-qPCR-RP1	TCTCCGGCACAAACTCAT
	AOC1-qPCR-FP	AGAACTTGGGAAATACCG
	AOC1-qPCR-RP1	GTTTGTGAATGGGACGAG
	AOC2-qPCR-FP	TCACTGCCAAGAAGAACC
	AOC2-qPCR-RP1	CTCCGAGACCGAACATTA
	AOC3-qPCR-FP	CTCGTAATCATCAATCTCAC
	AOC3-qPCR-RP1	ACTGCTGGACTGTTTCTAT
	OPR3-qPCR-FP	GGCGTTGGCAGAGTATTA
	OPR3-qPCR-RP1	AAACCTCCCTTAGCGTGA
	CYP94B3-qPCR-FP	ATTAGTGCTCGCCGTGCCA
	CYP94B3-qPCR-RP	TTGCTTTGCTTCTGCTCC
	JOX3-qPCR-FP	ATCTCCATTACCAGCCTTCG

	JOX3-qPCR-RP	ATCCACCTGATTCCTCTTTACC
	JAR1-qPCR-FP	CTCACTGGTCACCCTGTTCC
	JAR1-qPCR-RP	TCCCGTTGATATGTACTGCTTG
	JAZ1-qPCR-FP	CGGGCAAGTGATTGTATT
	JAZ1-qPCR-RP	GAGGATTGGATTGGCTCT
	JAZ2-qPCR-FP	AATGATGAATCTGTTCCCTT
	JAZ2-qPCR-RP	CACCATAACTCGACCACC
	JAZ5-qPCR-FP	CCTGATCTCAATGAGCCTAC
	JAZ5-qPCR-RP	CGATGATGACCTGCGTTT
	JAZ9-qPCR-FP	ACACTGCTGCTACCTCATCA
	JAZ9-qPCR-RP	CTCATAAGCCTCTCTTTGCG
	JAZ10-qPCR-FP	CGATTTCCTCGGACTTGA
	JAZ10-qPCR-RP	CTTCCCTCGGAGTAGACG
	MYC2-qPCR-FP	GAACGAAGATAAAGTTCTATCA
	MYC2-qPCR-RP	CAACCGCTCGTAACGCGTAGA
	GSL2- qPCR-FP	ACGAGAAGTTTGCAGAGAACTA
	GSL2- qPCR-RP	CTCTGCTGCTTTCCCATAAATC
	GSL5- qPCR-FP	CAAGCAATGAAGACTTCTACCG
	GSL5- qPCR-RP	ATTACTGAAGAAAGCAATCCGC
	GSL7- qPCR-FP	CCTTGGTTTCTTGTCAGTCATG
	GSL7- qPCR-RP	CTAATCCTTTGAACACGCTACG

	GSL9- qPCR-FP	ATTGCATGAGTTGTGCTGTTAG
	GSL9- qPCR-RP	TTGCAGCACATATTCAGTTAGC
	AT5G20330-qPCR-FP	ATCGAGTCTTACTTAGCTGACG
	AT5G20330-qPCR-RP	TAGGTTGGTTAGAGACTGCATG
	AT5G20340-qPCR-FP	TTGTTCTTCTTCTCGTGCATTG
	AT5G20340-qPCR-RP	CGTTAAGAACCTCAGTGTTTGG
	AT5G20390-qPCR-FP	CTATCCTCGTCAACATCTACCC
	AT5G20390-qPCR-RP	GTTGGTGTTGAAGATGGCATAG
	AT5G64790-qPCR-FP	AAACGTGACCCAGTATATTCGT
	AT5G64790-qPCR-RP	TGTGAATGTTCTTTAACGCTGG
	AT1G33220-qPCR-FP	AACCTTTACAAGTCCATAGGCA
	AT1G33220-qPCR-RP	GTGACTTCAATATTACGGTGGC
